# High expression of cAMP responsive element binding protein 1 (CREB1) is associated with metastasis, tumor stage and poor outcome in gastric cancer

**DOI:** 10.18632/oncotarget.3392

**Published:** 2015-03-18

**Authors:** Ya-Wen Wang, Xu Chen, Ji-Wei Gao, Hui Zhang, Ran-Ran Ma, Zu-Hua Gao, Peng Gao

**Affiliations:** ^1^ Department of Pathology, School of Medicine, Shandong University, Jinan, P.R. China; ^2^ Department of Pathology, McGill University, Montreal, Canada

**Keywords:** gastric cancer, CREB1, metastasis, prognosis, miRNA

## Abstract

cAMP responsive element binding protein 1 (CREB1) has been reported to be implicated in tumor development and progression of human cancers. However, the clinical significance and regulatory mechanisms of CREB1 expression in gastric cancer remain largely unknown. In the present study, immunohistochemistry was performed to detect the expression of CREB1 protein in 185 primary gastric cancer tissues, 50 secondary lymph node metastatic foci and 50 nontumorous gastric tissues. A prognostic model combining CREB1 expression with TNM tumor stage was constructed by logistic regression analysis. Regulation of CREB1 by miRNAs was investigated by luciferase reporter assay and Western blot. It was shown that CREB1 was highly expressed and correlated with lymph node metastasis, distant metastasis and tumor stage and poor outcome in gastric cancer. The prognostic model was proven to be an independent prognosis predictor and performed better than CREB1 or tumor stage alone. CREB1 was identified as a direct target of miR-27b and miR-200b, and down-regulated by miR-27b/miR-200b. We conclude that CREB1 is a promising biomarker to predict tumor metastasis and patient outcome in gastric cancer, and the miR-27b/miR-200b-CREB1 pathway may serve as a potential molecular target for the treatment of gastric cancer.

## INTRODUCTION

Gastric cancer is the fourth most common cancer worldwide and the second leading cause of cancer-related mortality in humans [1]. Despite the considerable improvement in cancer diagnosis and comprehensive therapy, patients with advanced gastric cancer still have poor prognosis due to tumor invasion and metastasis [2, 3]. The identification of precise factors driving the metastasis cascade and new biomarkers for prediction of prognosis is urgently needed to improve the early diagnosis and prognosis of patients with gastric cancer.

cAMP responsive element binding protein 1 (CREB1) is a well characterized transcription factor that belongs to the basic leucine zipper (bZIP) family [4]. As a transcriptional activator, CREB1 binds to the conserved cAMP-responsive element (CRE) on the promoter and mediates transcriptional responses to a variety of stimuli including neurotransmitters, hormones, membrane depolarization, and growth and neurotrophic factors, thereby acting as a mediator between different signal pathways and the downstream target-genes transcription [5, 6]. Intriguingly, mounting evidence suggests that CREB1 has potentially oncogenic functions and plays critical roles during carcinogenesis and cancer progression [7]. For example, CREB1 has been found to increase abnormal proliferation and survival of myeloid cells and to be associated with worse survival in patients with acute myeloid leukemia [8]. Tan et al. have shown that CREB1 could promote gliomagenesis by stimulating the expression of oncogenic microRNA-23a [9]. Recently, overexpression of CREB1 has been reported to be associated with poor prognosis in non-smokers with non-small cell lung cancer and in patients with breast cancer [10, 11]. However, the expression and clinicopathological significance of CREB1 in gastric cancer, especially the underlying mechanisms of CREB1 expression are still not well understood.

MicroRNAs (miRNA, miR) are a class of naturally occurring small noncoding RNAs that participate in the post-transcriptional regulation of gene expression by targeting the 3′ untranslated region (3′-UTR) of mRNAs, with either inducing mRNA degradation or blocking protein translation [12]. Emerging evidence has shown that miRNAs can function as tumor suppressors or oncogenes in the tumorigenesis and progression of various human cancers, including gastric cancer [13]. For example, microRNA-145 has been reported to be downregulated in gastric cancers and to suppress invasion-metastasis cascade by inhibiting N-cadherin and CTNND1 [14, 15]. MicroRNA-101 has been revealed to be reduced pronouncedly in metastatic cancers, and targeting EZH2 to decrease cell proliferation and motility [16]. Given the roles of miRNAs as regulators of gene expression in cancer development and progression, we hypothesized that they may play a part in modulating CREB1 expression.

In the present study, we demonstrate, for the first time, that CREB1 is overexpressed in gastric cancer and associated with poor outcome in patients with gastric cancer. Furthermore, our data suggest that CREB1 is directly targeted and inhibited by miR-27b and miR-200b.

## RESULTS

### CREB1 expression was stepwise increased in primary gastric cancer tissues and secondary lymph node metastatic foci, compared with nontumorous gastric mucosa

**Figure 1:**
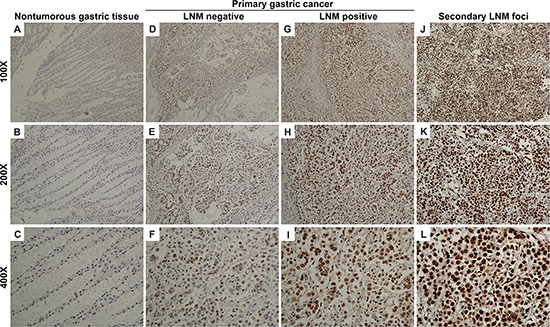
CREB1 expression in nontumorous gastric mucosa, primary gastric cancer tissues and secondary lymph node metastatic foci by immunohistochemistry **A–C.** Negative CREB1 expression in nontumorous gastric mucosa (only nuclear staining was considered in this study). **D–F.** Weak intensity with low positivity rate in primary gastric cancer tissues without lymph node metastasis. **G–I.** Weak to strong intensity with moderate positivity rate in primary gastric cancer tissues with lymph node metastasis. **J–L.** Strong intensity with high positivity rate in secondary lymph node metastatic foci.

**Figure 2:**
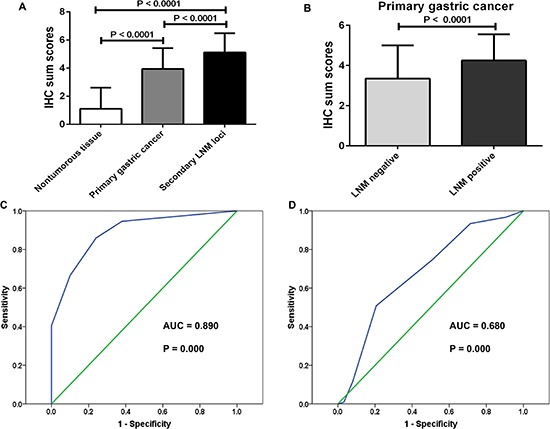
Upregualtion of CREB1 in more aggressive gastric tissues and ROC curves to assess the diagnostic value of CREB1 expression in gastric cancer **A.** IHC sum scores (0–7) were used to compare CREB1 expression in different gastric tissues. CREB1 was significantly elevated in primary gastric cancer tissues compared to nontumorous gastric mucosa (*t*-test, *P* < 0.0001). Further upregualtion of CREB1 was observed in secondary lymph node metastatic foci (*t*-test, *P* < 0.0001). **B.** CREB1 was dramatically overexpressed in primary gastric cancer tissues with lymph node metastasis than those without lymph node metastasis (*t*-test, *P* < 0.0001). **C.** The ROC curves reflected strong separation between gastric cancer tissues and nontumourous tissues, with an area under curve (AUC) of 0.890 (*P* = 0.000). **D.** To test the ability of CREB1 as a diagnostic marker for lymph node metastasis, ROC curves were established. Clear separation was observed between the patients with and without lymph node metastasis, with an AUC of 0.680 (*P* = 0.000).

Because CREB1 dysregulation in gastric cancer is still not well understood, we firstly investigated the expression of CREB1 protein by immunohistochemistry (IHC) in a total of 285 paraffin-embedded gastric samples including 185 cases of primary gastric cancer tissues, 50 cases of secondary lymph node metastatic foci and 50 cases of nontumorous gastric mucosa. Figure 1 represents the immunostaining profiles of CREB1 in gastric samples, with CREB1 staining predominantly observed in the nuclei of cells. These results provide clues that CREB1 mainly exerts its role as transcription factor in cell nuclei. CREB1 expression was negative or weak in nontumorous gastric tissues (Figure 1A–1C), whereas weak to strong expression was observed in primary gastric cancer tissues (Figure 1D–1I). Furthermore, even stronger expression was seen in secondary lymph node metastatic foci (Figure 1J–1L). In the 50 nontumorous gastric tissues, 31 (62.0%) cases showed negative CREB1 expression, 19 (38.0%) samples had weak expression, and none displayed strong expression (Table 1). In contrast, CREB1 immunoreactivity was predominantly identified as positive in the majority of primary gastric cancer tissues. Among the 185 primary gastric cancer tissues, 100 (54.1%) cases showed weak expression, 75 (40.5%) cases displayed strong expression and only 10 (5.4%) cases were classified as negative. In addition, in the 50 secondary lymph node metastatic foci, 16 (32.0%) cases showed weak expression, 34 (68%) cases displayed strong expression. Our data showed that CREB1 expression exhibited a gradual increase from nontumorous gastric mucosa via primary gastric cancer tissues, to secondary lymph node metastatic foci (Table 1, chi-square test; Figure 2A, *t*-test; *P* < 0.05). More interestingly, we found that CREB1 expression in cancerous tissues with lymph node metastasis (LNM) was significantly higher than that in cancerous tissues without LNM (Figure 1D–1I; Figure 2B, *t*-test; *P* < 0.05), suggesting that CREB1 may be associated with lymph node metastasis in gastric cancer.

**Table 1:** Expression of CREB1 protein in nontumorous gastric mucosa, primary gastric cancer tissues and secondary lymph node metastatic foci

Tissue samples	n	CREB1 expression			*P* value
		Negative (%)	Weak (%)	Strong (%)	
**Nontumorous gastric mucosa**	50	31 (62.0%)	19 (38.0%)	0 (0%)	< 0.0001^a^
**Primary gastric cancer tissues**	185	10 (5.4%)	100 (54.1%)	75 (40.5%)	< 0.0001^b^
**Secondary lymph node metastatic foci**	50	0 (0%)	16 (32.0%)	34 (68%)	0.0015^c^

a Difference between nontumorous gastric mucosa and primary gastric cancer tissues.

b Difference between primary gastric cancer tissues and secondary lymph node metastatic foci.

c Difference between nontumorous gastric mucosa and secondary lymph node metastatic foci.

In order to determine the diagnostic value of CREB1 in gastric cancer, receiver operator characteristic (ROC) curves were constructed and the area under the curve (AUC) was calculated to assess the ability of CREB1 expression (IHC sum scores) to differentiate between cancerous cases and nontumorous cases, or cancerous tissues with LNM and cancerous tissues without LNM. The ROC curves suggested that the AUC value for CREB1 to discriminate between gastric cancer tissues and nontumorous tissues was up to 0.890 (Figure 2C, CI (95%): 0.843–0.937, *P* = 0.000). Moreover, the AUC value for subgroups of gastric cancer tissues with LNM and those without LNM was 0.680 (Figure 2D, CI (95%): 0.596–0. 763, *P* = 0.000). Importantly, the estimated sensitivity, specificity, positive predictive value (PPV), and negative predictive value (NPV) of CREB1 expression to detect LNM were 79.4%, 50.8%, 45.5%, and 82.7%, respectively (Supplementary Table S1). These data indicated that the expression level of CREB1 was useful to predict the lymph node metastasis in patients with gastric cancer.

### CREB1 expression was correlated with lymph node metastasis, distant metastasis and tumor stage in primary gastric cancer

To further assess the clinical significance of CREB1 in gastric cancer, we analyzed the correlation between CREB1 expression and clinicopathological factors in primary gastric cancer (Table 2). CREB1 expression was found to be significantly positively correlated with lymph node metastasis (*P* = 0.0002), distant metastasis (*P* = 0.0007), and tumor stage (*P* = 0.008). However, no significant association was observed between CREB1 expression and age (*P* = 0.3996), gender (*P* = 0.6487), tumor size (*P* = 0.1548), depth of invasion (*P* = 0.5942), or tumor histological differentiation (*P* = 0.9583) (Table 2). All these data suggested an interesting link between CREB1 and gastric cancer metastasis and progression.

**Table 2:** Association between CREB1 expression and clinicopathological factors in primary gastric cancer

Variable	n	CREB1 expression			*P* value
		Negative	Weak	Strong	
**Age**					
≤ 60	92	6	53	33	
> 60	93	4	47	42	0.3996
**Gender**					
Male	152	8	80	64	
Female	33	2	20	11	0.6487
**Tumor size**					
≤ 4	82	2	48	32	
> 4	94	8	46	40	
Missing	9	0	6	3	0.1548
**Depth of invasion (T)**					
T1	16	2	9	5	
T2	78	5	41	32	
T3	67	3	34	30	
T4	19	0	13	6	
Missing	5	0	3	2	0.5942
**Lymph node metastasis (LNM)**					
Negative (N0)	63	6	44	13	
Positive (N1–N3)	122	4	56	62	0.0002
**Distant metastasis (M)**					
Negative (M0)	131	8	81	42	
Positive (M1)	49	2	16	31	
Missing	5	0	3	2	0.0007
**Tumor stage^1^**					
I	39	5	27	7	
II	37	0	18	19	
III	49	3	28	18	
IV	55	2	24	29	
Missing	5	0	3	2	0.008
**Differentiation**					
Well	8	0	5	3	
Moderate	64	4	35	25	
Poor	111	6	60	45	
Missing	2	0	0	2	0.9583

1 Stages IA and IB are regarded as stage I, and stages IIIA and IIIB as stage III.

### CREB1, especially the prognostic model combining CREB1 expression and tumor stage, could serve as a prognostic biomarker indicating poor survival in patients with gastric cancer

**Figure 3:**
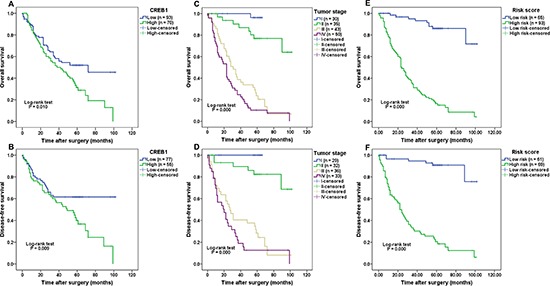
Correlation of CREB1 expression with survival of patients with gastric cancer **A–B.** Patients with high CREB1 expression had significantly poorer overall survival (*P* = 0.010) and disease-free survival (*P* = 0.009) than those with low CREB1 expression. **C–D.** Advanced tumor stage was indicative of worse prognosis (OS and DFS, both *P* = 0.000) in patients with gastric cancer. **E–F.** A prognostic model combining CREB1 expression with tumor stage was constructed. Patients in the high-risk group displayed shorter survival time (OS and DFS, both *P* = 0.000) than those in the low-risk group.

As CREB1 expression was significantly overexpressed and correlated with aggressive clinical characteristics in gastric cancer, we further evaluated the association of CREB1 expression with the prognosis of gastric cancer patients. Given the limited sample size of patients in the CREB1 negative expression group, we combined the patients in negative expression group and weak expression group, and defined these as low expression group. In addition, strong CREB1 expression was considered as high expression group. The survival analysis showed that patients with high expression of CREB1 displayed a significantly poorer overall survival (OS) and disease-free survival (DFS) than those who had low CREB1 expression (Figure 3A, 3B, *P* = 0.010 and *P* = 0.009 respectively). The estimated sensitivity, specificity, PPV, and NPV of high CREB1 expression to predict death of patients were 74.7%, 55.3%, 55.8% and 74.3%, respectively (Supplementary Table S2). As expected, we found that several well-known prognosis-related factors, including larger tumor size, deeper tumor invasion, positive LNM, distant metastasis and advanced tumor stage, were all indicative of worse prognosis in the current set of patients (Supplementary Figure S1; Figure 3C–3D; *P* < 0.05). This result validated the efficacy of our experimental system.

Univariate and multivariate analysis by the Cox proportional hazards regression model were used to explore factors associated with patient outcome. The univariate analysis suggested that CREB1 expression, tumor size, depth of invasion, lymph node metastasis, distal metastasis and tumor stage were significantly correlated with OS and DFS of gastric cancer patients (Table 3, *P* < 0.05). Multivariate analysis showed that CREB1 expression was not an independent prognostic predictor for OS and DFS (*HR* = 1.487, CI (95%): 0.926–2.388, *P* = 0.101 and *HR* = 1.400, CI (95%): 0.804–2.436, *P* = 0.234), however, it still supports CREB1 as a risk factor (*HR* > 1) for patient survival. In our study, tumor stage was confirmed as an independent prognostic factor (*HR* = 2.062, CI (95%): 1.317–3.230, *P* = 0.002 and *HR* = 2.227, CI (95%): 1.258–3.943, *P* = 0.006 for OS and DFS respectively) for patients with gastric cancers.

**Table 3:** Univariate and multivariate analysis for overall survival and disease-free survival after surgery (Cox proportional hazards regression model)

Variable	Univariate analysis			Multivariate analysis		
	HR	CI (95%)	*P* value	HR	CI (95%)	*P* value
**Overall survival**						
CREB1 expression	1.692	1.125–2.534	0.011	1.487	0.926–2.388	0.101
Tumor size	2.191	1.424–3.371	0.000	1.436	0.901–2.289	0.128
Depth of invasion	1.585	1.244–2.019	0.000	1.160	0.780–1.723	0.464
Lymph node metastasis	4.906	2.712–8.878	0.000	1.894	0.950–3.776	0.070
Distal metastasis	4.450	2.869–6.903	0.000	1.712	0.795–3.685	0.169
Tumor stage	2.777	2.157–3.575	0.000	2.062	1.317–3.230	0.002
Differentiation	1.465	1.010–2.127	0.044	1.162	0.770–1.752	0.475
Risk score	11.713	5.874–23.461	0.000	6.529	1.964–21.70	0.002
**Disease-free survival**						
CREB1 expression	1.907	1.159–3.137	0.011	1.400	0.804–2.436	0.234
Tumor size	2.139	1.283–3.566	0.004	1.331	0.762–2.325	0.315
Depth of invasion	1.977	1.478–2.646	0.000	1.174	0.715–1.926	0.527
Lymph node metastasis	5.185	2.543–10.572	0.000	1.690	0.731–3.911	0.220
Distal metastasis	4.014	2.294–7.022	0.000	1.393	0.542–3.579	0.492
Tumor stage	2.998	2.217–4.053	0.000	2.227	1.258–3.943	0.006
Risk score	14.457	6.198–33.720	0.000	8.036	1.923–33.57	0.004

Next we asked whether CREB1 expression could improve the prognostic value of tumor stage, therefore a prognostic model combining CREB1 expression with tumor stage was constructed by logistic regression [17]. The coefficients (±standard error) of CREB1 expression and tumor stage were 2.011 (±0.567) and 2.342 (±0.374), respectively, with the constant of –6.740 (±1.135). Thus the prognostic model was built as follows: Risk score = 2.011 × CREB1 expression + 2.342 × tumor stage –6.740, where the definition was as follows: for CREB1 expression (0 = low expression and 1 = high expression), and for tumor stage (I = 1, II = 2, III = 3 and IV = 4) in each patient. The patients were divided into high-risk and low-risk groups using the median risk score as the cut-off point. The survival analysis showed that patients in the high-risk group had significantly poorer OS and DFS, compared with those in the low-risk group (Figure 3E, 3F, *HR* = 11.71, *P* = 0.000 and *HR* = 14.45, *P* = 0.000 for OS and DFS respectively). Moreover, multivariate analysis showed that the risk score was an independent prognostic factor (*HR* = 6.529, CI (95%): 1.964–21.70, *P* = 0.002 and *HR* = 8.036, CI (95%): 1.923–33.57, *P* = 0.004). Given the relatively higher HR (hazard ratio) and clearer separation between high-risk and low-risk groups in the survival curves (Supplementary Table S3), we suggest that the prognostic model was more effective than CREB1 expression or tumor stage alone to predict patients' outcome.

### CREB1 expression was inhibited by miR-27b and miR-200b

In order to investigate whether miRNAs could regulate aberrant CREB1 expression in gastric cancer, we used prediction algorithms such as miRWalk [18] and starBase [19] to screen the miRNAs that potentially target CREB1. These two algorithms could integrate miRNA-targets interactions information produced by several established miRNA prediction programs i.e. RNA22, miRanda, miRDB, TargetScan, RNAhybrid, and Diana-microT. Based on these data and the previous reports about the candidate miRNAs' function, we chose 5 cancer-related or tumor-suppressing miRNAs, including miR-214, miR-200b, miR-27b, miR-32, and miR-429, for further investigation.

**Figure 4:**
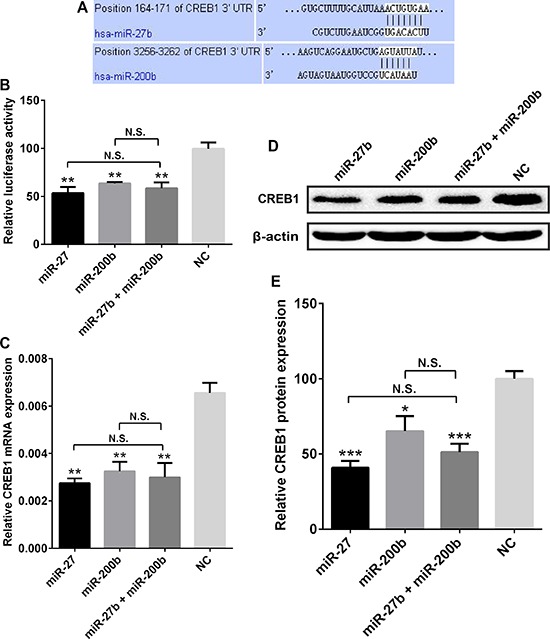
CREB1 expression was inhibited by miR-27b and miR-200b **A.** Sequence alignment of miR-27b and miR-200b with the 3′-UTR of CREB1. **B.** miR-27b, miR-200b, and miR-27b/miR-200b co-transfection could suppress the luciferase activity in pmirGLO-CREB1 transfected SGC7901 cells by 46.29 ± 8.20% 36.06 ± 3.07%, and 41.14 ± 7.80% respectively (*P < 0.05, **P < 0.01, ***P < 0.001, N.S. = nonsignificant). **C.** Compared with negative control, miR-27b, miR-200b, and miR-27b/miR-200b co-transfection could significantly reduced the CREB1 mRNA expression in SGC7901 cells by 57.81 ± 5.74%, 49.98 ± 9.29% and 53.92 ± 11.2% respectively (*P < 0.05, **P < 0.01, ***P < 0.001, N.S. = nonsignificant). **D, E.** miR-27b, miR-200b, and miR-27b/miR-200b co-transfection led to dramatic reduction of CREB1 protein expression in SGC7901 cells with the decrease of 59.20 ± 2.46%, 34.77 ± 8.94% and 48.76 ± 4.49% respectively (*P < 0.05, **P < 0.01, ***P < 0.001, N.S. = nonsignificant).

The luciferase assays revealed that miR-27b and miR-200b (Figure 4, Supplementary Figure S3), rather than miR-214, miR-32, and miR-429 (Supplementary Figure S2, *P* > 0.05) could significantly suppress the luciferase activity in pmirGLO-CREB1 (3′-UTR) and miRNAs co-transfected cells. Specifically, miR-27b and miR-200b transfection led to 46.29 ± 8.20% and 36.06 ± 3.07% decrease of luciferase activity in SGC7901 cells respectively (Figure 4A, 4B, *P* = 0.0016 and 0.0054). To test whether miR-27b and miR-200b decreased CREB1 expression at mRNA level, we detected the CREB1 mRNA expression in gastric cancer cells transfected with miR-27b/miR-200b. We found that miR-27b and miR-200b could dramatically reduce the CREB1 mRNA expression by 57.81 ± 5.74% and 49.98 ± 9.29% respectively in SGC7901 cells (Figure 4C, *P* = 0.0025 and 0.0011). Furthermore, Western blot analysis validated that miR-27b and miR-200b could significantly inhibit the expression of CREB1 protein in SGC7901 cells, with the decrease of 59.20 ± 2.46% and 34.77 ± 8.94% respectively (Figure 4D, 4E, *P* = 0.0003 and 0.0165). Inhibition of miR-27b and miR-200b on CREB1 expression was also seen in MKN45 cells (Supplementary Figure S3). These data suggest that CREB1 is a direct target of miR-27b/miR-200b, and is downregulated by miR-27b/miR-200b.

Subsequently, we tested whether miR-27b and miR-200b could synergistically inhibit CREB1 expression in gastric cancer. The gastric cancer cells were co-transfected with miR-27b (15 nM) and miR-200b (15 nM), and subjected to luciferase assay and CREB1 expression detection. We found that miR-27b and miR-200b co-transfection led to significant decrease of luciferase activity (41.14 ± 7.80%), CREB1 mRNA expression (53.92 ± 11.2%) and CREB1 protein level (48.76 ± 4.49%) in SGC7901 cells (Figure 4, *P* < 0.05). However, miR-27b/miR-200b co-transfection did not show stronger ability than miR-27b or miR-200b alone (Figure 4, *P* > 0.05). For example, compared with the negative control, miR-27b, miR-200b and miR-27b/miR-200b co-transfection reduced the protein level of CREB1 by 59.20 ± 2.46%, 34.77 ± 8.94% and 48.76 ± 4.49% respectively. Although miR-27b/miR-200b co-transfection seemed to show more power than miR-200b in suppressing the expression of CREB1, it displayed less activity than miR-27b. Therefore we could not conclude that miR-27b and miR-200b have synergistic roles in inhibiting CREB1 expression in gastric cancer.

## DISCUSSION

An increasing number of studies have shown that CREB1 was aberrantly expressed in a number of human cancers including both solid tumors [9, 11, 20, 21] and hematological malignancy [8, 22, 23]. Son et al. have found that CREB1 was overexpressed in metastatic breast cancer cells than non-metastatic ones, and promoted breast cancer metastasis and subsequent bone destruction [20]. CREB1 has also been found to be highly expressed in glioma tissues and enhanced glioma cell growth survival by inducing the expression of oncogenic microRNA-23a [9]. However, there is still evidence showing that CREB1 suppresses the glioblastoma proliferative effect of the stress-induced acetylcholinesterase variant AChE-R [21], suggesting a controversial or tissue-specific role of CREB1 in human cancers.

Although CREB1 has been extensively investigated in various tumors [11, 24], the evaluation methods for CREB1 immunohistochemistry (IHC) remain underdeveloped. Here we explored a semi-quantitatively scoring criterion in IHC evaluation for CREB1, considering both the staining intensity and percentage of positive staining. The subsequent verifications, including ROC curves, clinical factors-related analysis and survival analysis, confirmed that the present evaluation method is appropriate for CREB1 analysis. Of note, the optimal cut-off points 2 and 4 (IHC sum score), which were used in our classification of subgroups, were further validated in ROC analysis (data not shown), with the lowest 95% confidence interval (for sensitivity and specificity) > 50% and the *P*-value < 0.05. Importantly, CREB1 determination by IHC in the present experimental and evaluation system could distinguish lymph node metastasis in patients with gastric cancer and predict patients' prognosis.

Kong et al. have demonstrated that the expression levels of CREB1 mRNA in 10 cases of gastric adenocarcinoma tissues was significantly higher than that in the matched normal tissues, and they found that CREB1 promoted MGC-803 cell proliferation [25]. Reports on the relationship between CREB1 and gastric cancer metastasis remain scarce. In the current study, we found that CREB1 was overexpressed in gastric cancer tissues, in comparison with nontumorous gastric mucosa. Interestingly, our data demonstrated that CREB1 was further elevated in secondary lymph node metastatic foci, suggesting an interesting link between CREB1 and lymph node metastasis of gastric cancer. Moreover, our results showed that CREB1 is positively related with lymph node metastasis, distant metastasis and tumor stage in primary gastric cancer. Consistent with our findings, Chhabra et al. reported that node-positive breast tumors had higher levels of CREB1 than node-negative tumors [11]. More recently, Son et al. found that metastatic MDA-MB-231 breast cancer cells exhibited higher CREB1 expression than non-metastatic MCF-7 cells [20].

To establish the prognostic value of CREB1 in patients with gastric cancer, survival analysis was performed. The current study reports, for the first time, that high expression of CREB1 was indicative of poor prognosis in gastric cancer. In addition, univariate and multivariate Cox regressions further confirmed CREB1 as a risk factor (HR > 1) for patients with gastric cancer, though CREB1 seemed to bear no statistical significance in multivariate analysis. Consistently, CREB1 was previously found to be an unfavorable prognostic factor for patients with non-small cell lung cancer, breast cancer and hepatocellular carcinoma [10, 11, 24]. These results suggest that CREB1 may be a valuable biomarker in predicting the prognosis of human cancers.

It is generally accepted that TNM tumor stage is the most important prognostic determinant [26] for cancer patients. However, it still has several limitations in clinical practice [27, 28]. For example, some subgroups of the TNM classification did not have significantly different survival rates [27, 29]. Thus the prognostic model, which has been used in previous studies [17], was constructed in an attempt to test whether CREB1 expression could improve the predictive power of the conventional TNM tumor stage. As expected, the prognostic model is a more powerful predictor than CREB1 expression or tumor stage alone. We observed clearer separation between high-risk and low-risk subgroups of the prognostic model in the survival curve. And, compared with CREB1 and tumor stage, risk score showed higher HR (hazard ratio) in stratifying patients with different prognosis.

Although CREB1 has been shown to be aberrantly expressed in several human cancers, information about its regulation is relatively unclear. In this study we showed that CREB1 was a target of miR-27b and miR-200b, and inhibited by miR-27b/miR-200b in both mRNA and protein levels. MiR-27b and miR-200b have been reported to be downregualted in numerous human tumors, including gastric cancer [30–33]. In these previous studies, decreased expression of miR-27b/miR-200b was identified as an unfavorable prognostic factor and miR-27b/miR-200b reduced cellular proliferation, migration and invasion, suggesting potentially tumor-suppressing roles of miR-27b/miR-200b in human cancers. Interestingly, Yang et al. have shown that microRNA-433 inhibition of CREB1 expression repressed cell migration in hepatocellular carcinoma [34]. These findings highlighted the critical role of miRNAs in regulating CREB1 expression. In this study we found that miR-27b and miR-200b inhibited CREB1 expression in gastric cancer. This suggested to us that aberrant overexpression of CREB1 in gastric cancer may be partially due to the downregulation of miR-27b/miR-200b in gastric cancer, and miR-27b/miR-200b could be potential CREB1 inhibitors to suppress carcinogenesis and tumor progression. Recently, miR-200b and miR-22 have been shown to synergistically suppress Wnt-1 in gastric cancer, indicating an additive effect of miRNAs in modulating gene expression via a fine-tuning manner [35]. However, in the present study, we did not observe synergistic action of miR-27b and miR-200b in inhibiting CREB1 expression in gastric cancer. We suspect that this may be due to the fact that miR-27b has already strongly inhibited the expression of CREB1 by ~50% or more in gastric cancer cells; therefore, it's hard to see evidently stronger inhibitory effect of miR-27b/miR-200b co-transfection on CREB1 expression. However, other uncharacterized mechanisms underlying the regulation of miR-27b and miR-200b on CREB1 expression still need further investigation.

Collectively, our data demonstrated that CREB1 was highly expressed and correlated with metastasis, tumor stage in gastric cancer. High expression of CREB1 was associated with poor outcome in gastric cancer patients. The prognostic model, combining CREB1 expression and tumor stage, displayed more effectiveness than either CREB1 expression or tumor stage alone to predict patients' survival. In addition, we identified a regulatory mechanism of CREB1 expression that was inhibited by miR-27b and miR-200b. Our findings suggest that CREB1, as a valuable biomarker of gastric cancer prognosis, may be a promising approach to gastric cancer treatment through the miR-27b/miR-200b-CREB1 pathway.

## MATERIALS AND METHODS

### Tissue samples

A total of 285 human gastric specimens were collected from Qilu Hospital of Shandong University. They included 185 cases of primary gastric carcinoma (among them 122 cases had lymph node metastasis), 50 cases of secondary lymph node metastatic foci and 50 cases of nontumorous gastric mucosa adjacent to carcinomas. The World Health Organization (WHO) classification (2000) and UICC/AJCC TNM classification (the 6th edition) was followed in pathological classification and tumor stage definition. All samples were fixed in 40g/L formaldehyde and embedded in paraffin for histological diagnosis and immunohistochemistry study. The patients were followed up clinically with median follow-up time of 44 months. The study was approved by the Ethics Committee of Shandong University, China. Informed consent was obtained from each subject.

### Immunohistochemistry

The streptavidin-peroxidase-biotin (SP) immu nohistochemical method was performed as previously described [14, 36]. Briefly, paraffin-embedded tissue sections were cut and immunostained with antibodies against CREB1 (Abcam, Cambridge, UK, dilution 1:350). For negative controls, the primary antibody was replaced with PBS.

### Evaluation of immunohistochemical staining

The immunohistochemical staining was evaluated in blind fashion by two experienced pathologists. For each sample, five hundred cells from three randomly chosen fields were counted. Staining was semi-quantitatively scored based on both the staining intensity (0 = negative; 1 = weak intensity; 2 = moderate intensity; 3 = strong intensity) and percentage of positively stained cells (0 = 0%, 1 = 1–25%, 2 = 26–50%, 3 = 51–75%, and 4 = 76–100%). The cut-off levels of the sum of scores were defined as follows: 0–1, negative expression, 2–4, weak expression, and 5–7, strong expression. The appropriateness of the cut-off points was further confirmed by receiver operating characteristics (ROC) curve analysis.

### Cell culture and transfection

The human gastric cancer cell lines SGC7901 (moderately-poorly differentiated) and MKN45 (poorly differentiated) were obtained from the American Type Culture Collection (Manassas, VA, USA). The cells were maintained in RPMI 1640 culture medium supplemented with 10% FBS in a humidified cell incubator with an atmosphere of 5% CO_2_ at 37°C. For cell transfection, exponentially growing cells (1.5 × 10^5^) were seeded in 12-well plates and transfected with 30 nM miRNA mimics or the negative control (GenePharma, Shanghai, China) using the X-tremeGENE transfection reagent (Roche Applied Science, Indianapolis, IN, USA) according to the manufacturer's instructions.

### RNA isolation and real-time quantitative PCR

Total RNAs were extracted from transfected cells using Trizol Agent (Invitrogen, Carlsbad, CA, USA) and were reverse transcribed into cDNA using a Rever Tra Ace qPCR RT Kit (Toyobo, Osaka, Japan). Real-time quantitative PCR (RT-qPCR) was performed in a total volume of 10-μl SYBR Green Real-time PCR Master Mix (Roche Diagnostic GmbH, Mannheim, Germany) by a Bio-Rad CFX^TM^ 96 C1000 Real-Time system. All reactions were performed in duplicate. The mRNA expression levels of CREB1 were normalized to GAPDH mRNA levels using the 2^−ΔCT^ method. Primers of CREB1 for RT-qPCR were synthesized (GenePharma, Shanghai, China) as follows [37]: Forward 5′-GCTGCCTCTGGAGACGTACAA-3′, Reverse 5′-GCTAGTGGGTGCTGTGCGA-3′.

### Luciferase reporter assay

The pmirGLO miRNA target expression vector (Promega, San Lius Obispo, CA, USA) was used to construct the recombinant plasmid pmirGLO-CREB1 containing the CREB1 mRNA 3′-UTR fragments as previously described [14]. For the luciferase reporter assay, cells were co-transfected with 30 nM of miRNA mimics or negative control and 30 ng pmirGLO-CREB1 (3′-UTR) using Lipofectamine 2000 (Invitrogen, Carlsbad, CA, USA). Forty-eight hours after transfection, the luciferase activity was measured using the dual luciferase assay system (Promega, Madison, WI, USA) according to the manufacturer's instructions. Each experiment was performed in triplicate.

### Western blot assay

After 48 h transfection with miRNA mimics or negative control, cells were subjected to protein extraction using RIPA lysis buffer and protein concentration was quantified by a bicinchoninic acid protein assay kit (Beyotime Institute of Biotechnology). Then 50 ug of proteins were separated by electrophoresis on 8% SDS-polyacrylamide gel electrophoresis (SDS-PAGE), transferred to a polyvinylidene difluoride (PVDF) membrane (Millipore, Billerica, MA, USA) and blocked with 5% fat-free milk powder in TBS at room temperature for 2 h. Subsequently, the membrane was incubated with primary antibodies against CREB1 (rabbit monoclonal antibody, Abcam, dilution 1:1500) or β-actin (mouse polyclonal antibody, internal control, Zhongshan Goldenbridge Biotechnology, Beijing, China, dilution 1:2000) overnight at 4°C, washed, and then incubated with a horseradish peroxidase (HRP)-conjugated goat anti-rabbit or anti-mouse IgG secondary antibodies respectively at 37°C for 1 h. Immunoreactivity were visualized using an enhanced chemiluminescence kit (Millipore, Billerica, MA, USA) according to the manufacturer's instruction.

### Statistical analysis

Statistical analysis was performed using SPSS 18.0 (SPSS, Chicago, IL, USA) and GraphPad Prism 5 (GraphPad Software, Inc., San Diego, CA, USA). Student's *t* test was used to analyze the differences between two groups. The chi-square test was used to analyze the relationship between CREB1 expression and clinicopathological variables. The survival rates were calculated by the Kaplan-Meier method and the differences between the subgroups were examined by the log-rank test. The prognostic value of CREB1 expression was determined by univariate and multivariate analysis based on the Cox proportional hazards regression model. Variables with *P* values less than 0.05 by univariate analysis were then put into subsequent multivariate analysis. The logistic regression was used to build a prognostic model combining CREB1 expression with tumor stage. Receiver operating characteristics (ROC) curve analysis was performed to assess the diagnostic value of CREB1 expression in gastric cancer. *P* < 0.05 was considered to be statistically significant.

## SUPPLEMENTARY FIGURES AND TABLES


