# Supplementary material for: High expression of cAMP responsive element binding protein 1 (CREB1) is associated with metastasis, tumor stage and poor outcome in gastric cancer
Source: Oncotarget. 2015 Mar 18;6(12):10646–57. doi: 10.18632/oncotarget.3392 (PMC4496382; doi:10.18632/oncotarget.3392)
Supplement: Supplementary file 1 [file oncotarget-06-10646-s001.pdf]

SUPPLEMENTARY FIGURES AND TABLES

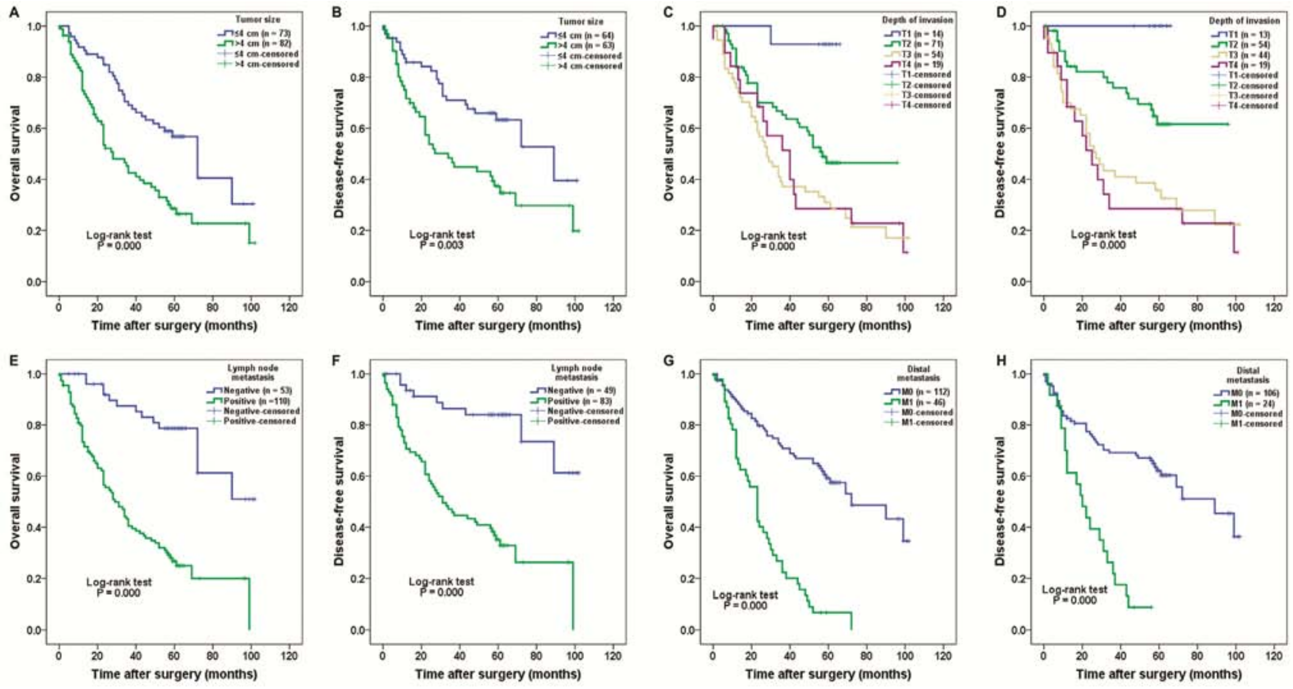

Supplementary Figure S1: Related to Figure 3. Kaplan–Meier plots shows the correlation of tumor size **A, B.** depth of invasion **C, D.** lymph node metastasis **E, F.** and distant metastasis **G, H.** with overall survival (OS) and disease-free survival (DFS) of patients.

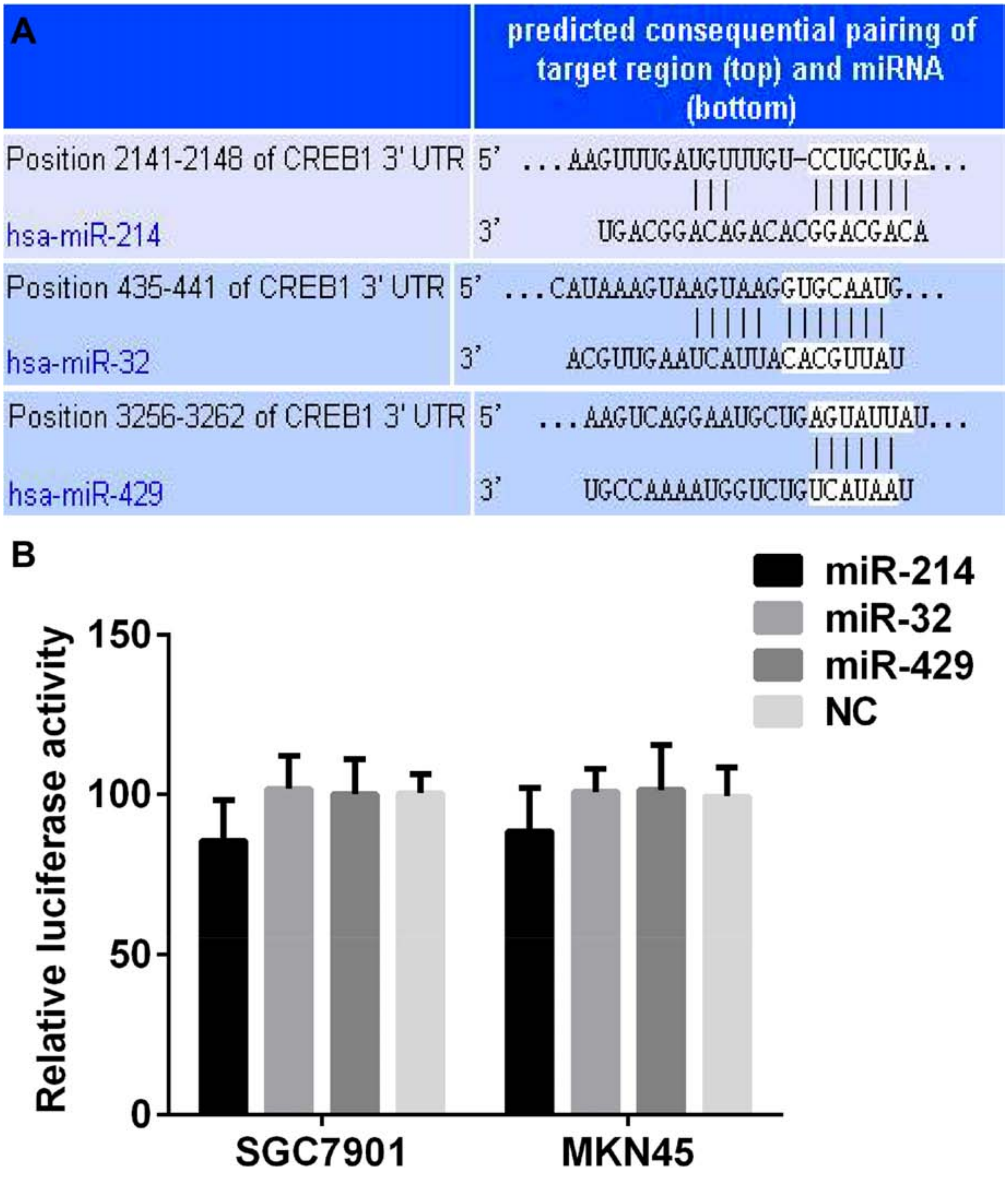

Supplementary Figure S2: Related to Figure 4. MiR-32 and miR-429 demonstrated no effect the luciferase activity in pmirGLO-CREB1 (3′-UTR) and miRNAs co-transfected SGC7901 and MKN45 cells. MiR-214 could decrease the relative luciferase activity to some degree (17% approximately), however, it displayed less activity than miR-27b and miR-200b.

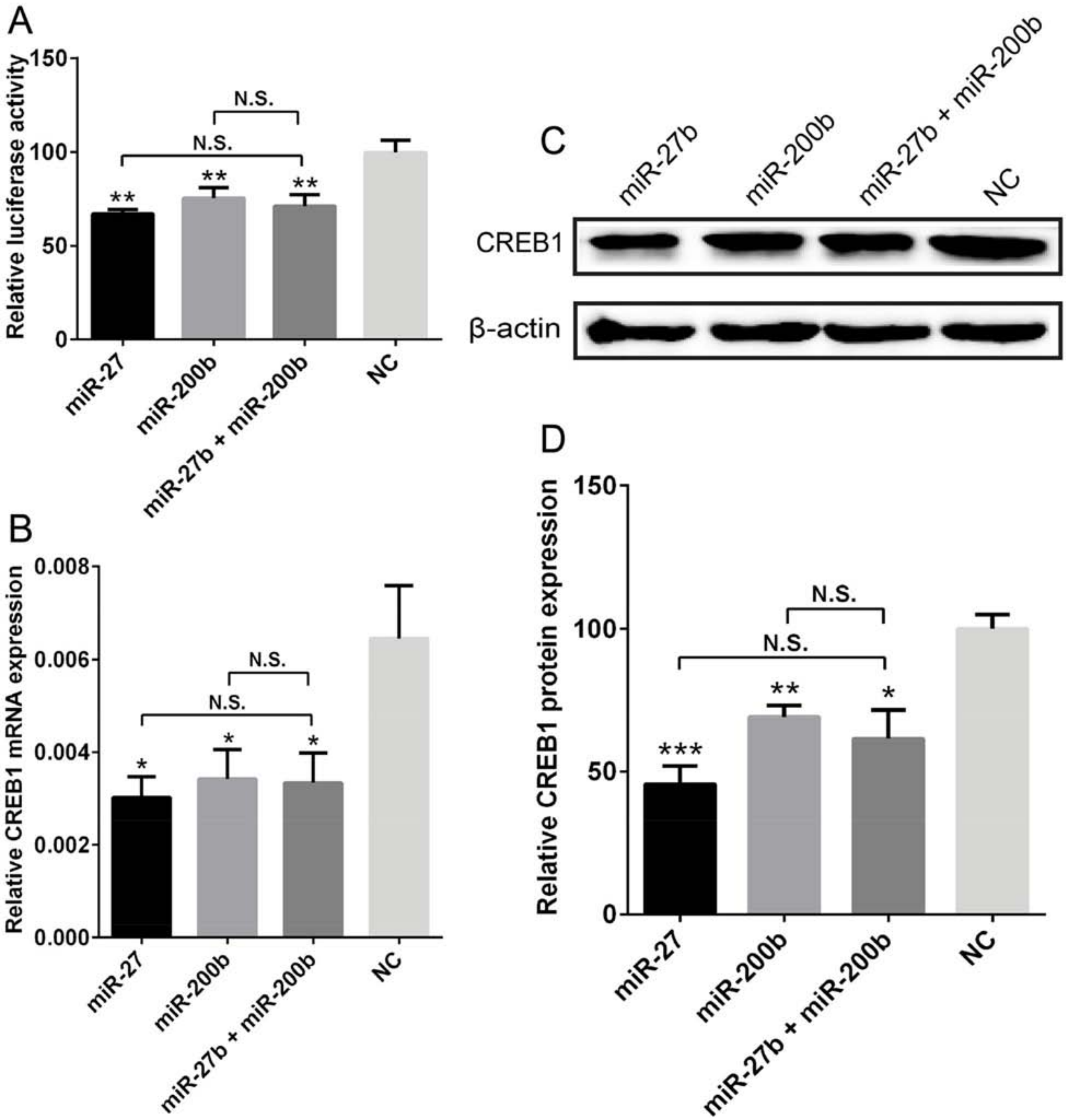

Supplementary Figure S3: Related to Figure 4. MiR-27b, miR-200b, and miR-27b/miR-200b co-transfection could significantly inhibit the luciferase activity **A.** CREB1 mRNA level **B.** and CREB1 protien level **C, D.** in MKN45 cells (*$P < 0.05$, **$P < 0.01$, ***$P < 0.001$, N.S. = nonsignificant).

Supplementary Table S1. Sensitivity, Specificity, and Positive and Negative Predictive Values for LNM detection using CREB1 expreesion

CREB1 expression	LNM[a] negative	LNM positive	Sensitivity	Specificity	PPV[b]	NPV[c]
**Low[d]**	50	60	79.4%	50.8%	45.5%	82.7%
**High[e]**	13	62				

[a]LNM = Lymph node metastasis
[b]PPV = Positive Predictive Value
[c]NPV = Negative Predictive Value
[d]According to the definition of Sensitivity, Specificity, and PPV and NPV, and given the limited sample size of CREB1 negative expression group, we classified the negative expression and weak expression as low expression group.
[e]Strong CREB1 expression was considered as high expression group.

Supplementary Table S2. Sensitivity, Specificity, and Positive and Negative Predictive Values for death detection using CREB1 expreesion

CREB1 expression	Survival[a]	Death	Sensitivity	Specificity	PPV	NPV
**Low**	50	60	74.7%	55.3%	55.8%	74.3%
**High**	13	62				

[a]Based on the clinical outcome, we divided the patients into survival group and death group.

Supplementary Table S3. Comparison of the effect of CREB1 expression, tumor stage and risk score on patients' survival

Covariates	Mean survival time (months)	HR	CI (95%)	*P* value
**CREB1 expression**	Low : High = 61 : 45	1.692	1.125–2.534	0.011
**Tumor stage**	I : II : III : IV = 65 : 85 : 35 : 28	2.777	2.157–3.575	0.000
**Risk score**	Low risk : High risk = 91 : 33	11.713	5.874–23.461	0.000
